# Stem Cell Niche Concept: Search for Current Expert Consensus

**DOI:** 10.3390/ijms26178422

**Published:** 2025-08-29

**Authors:** Igor Khlusov, Larisa Litvinova, Anastasia Efimenko

**Affiliations:** 1 Department of Morphology and General Pathology, Siberian State Medical University, 634050 Tomsk, Russia; khlusov63@mail.ru; 2 Center for Immunology and Cellular Biotechnology, Immanuel Kant Baltic Federal University, 636016 Kaliningrad, Russia; larisalitvinova@yandex.ru; 3 Medical Research and Educational Institute, Lomonosov Moscow State University, 119234 Moscow, Russia

**Keywords:** mammals, stem cell niche, hematopoietic stem cells, mesenchymal stromal/stem cells, bone marrow microenvironment, hierarchy, expert consensus

## Abstract

Postnatal stem cells are crucial for tissue homeostasis and repair and are regulated by specialized microenvironmental microterritories known as “stem cell niches”. Proposed by R. Schofield in 1978 for hematopoietic stem cells, niches maintain self-renewal, guide differentiation and maturation, and can even revert progenitor cells to an undifferentiated state. Niches respond to injury, oxygen levels, mechanical cues, and signaling molecules. While the niche concept has advanced regenerative medicine, bioengineering, and 3D bioprinting, further progress is hindered by inconsistent interpretations of its core principles. To address this, we proposed a consensus-building initiative among experts in regenerative medicine and bioengineering. We have developed a questionnaire covering the niche topography, hierarchy, dimension, geometry, composition, regulatory mechanisms, and specifically the mesenchymal stem cell niches. This pilot survey, being conducted under the auspices of the National Society for Regenerative Medicine in the Russian Federation, aims to establish a standardized framework on the eve of the 50th anniversary of Schofield’s hypothesis. The resulting consensus will guide future research and innovation in this pivotal field.

## 1. Introduction

Accumulating data indicates that postnatal stem cells play a central role in tissue homeostasis, repair, and regeneration. Stem cells require a favorable and instructive microenvironment in order to be well controlled. The concept of the stem cell niche was proposed almost 50 years ago by R. Schofield (1978) for hematopoietic stem cells (HSCs) and later further developed by others who have claimed that a niche can maintain the self-renewal of stem cells and stimulate their differentiation as well as the return of progenitor cells to an undifferentiated state. As part of the regulation of stem cell fate, tissue-specific niches should respond to injury and sense microenvironmental changes such as oxygenation, position, mechanotransduction, and multiple secreted factors that mediate cell-to-cell communication. The theory of stem cell niches has not only made a significant contribution to basic research in the field of regeneration but also provides the technological basis for the practical implementation of innovative approaches in regenerative medicine, tissue bioengineering, 3D bioprinting, microfluidic technologies, etc. However, although several more or less comprehensive reviews and experimental studies have been published in recent years demonstrating the progress made in stem cell niche research, a certain stagnation can be observed in this field, mainly due to the different and broad interpretations of the fundamental issues of the “niche” concept. We propose that this problem can be partially overcome by building a consensus among experts in regenerative medicine, developmental biology, and bioengineering. To this end, we have developed a questionnaire with six sections addressing the topography, hierarchy, and size of the stem cell niche; the contribution of different niche components to the regulatory function; the control of cell behavior within the niche; and the characteristics of stem cell niches of mesenchymal origin.

In this focused review, we discuss the key problems and current achievements in the development of the niche concept, present the questionnaire, and justify the wording of the statements to be interpreted and approved by the selected experts.

## 2. General Concept (Definition) of the Stem Cell Niche

A pivotal role of tissue-specific stem cells in tissue renewal and regeneration has been actively investigated for several decades [1,2,3,4]. The discussion about the inner mechanisms of stem cell regulation has been ongoing for the past 60 years [5]; the theory of hemopoietic-inductive microenvironment (HIM) [6] and the stochastic model (hemopoiesis engendered randomly, HER) [7,8] are among the main speculations of bone marrow (BM) research.

Within the HIM theory, an intriguing section is the HSC niche in adult mammals, formulated almost 50 years ago by Robert Schofield [9] and later further developed by other researchers who have claimed that a niche can maintain stem cell self-renewal and stimulate their differentiation as well as the return of progenitor cells to an undifferentiated state. According to Schofield’s own opinion, the “stem cell niche” hypothesis was introduced to explain the dependence of stem cells on their microenvironment [10].

Although many intrinsic and extrinsic factors (niche components) of HIM have been identified in the regulation of HSC origination, expansion, migration, and localization, the underlying mechanisms remain largely unknown [11]. In the context of stem cell fate control, tissue-specific niches should respond to injury and sense microenvironmental changes such as oxygenation, position, mechanotransduction, and a variety of secreted factors that mediate cell-to-cell communication. The specific microenvironment, or the stem cell niche, demands the presence of certain niche components that can maintain the stem cell pool and restore the microenvironment in injured tissues for their subsequent appropriate functioning. Sagaradze et al. (2020) and others consider that this role belongs to mesenchymal stromal/stem cells (MSCs) [12].

The evolving theory of stem cell niches has not only made a significant contribution to basic research in the field of regeneration but also provides the technological basis for the practical implementation of innovative approaches in regenerative medicine, tissue bioengineering, 3D bioprinting, microfluidic technologies, etc. In this regard, a query ‘cell niche’ showed 33,385 results generated by the text retrieval engine PubMed (https://pubmed.ncbi.nlm.nih.gov/; accessed on 7 May 2025) since 1956. In turn, the key phrase ‘stem cell niche’ was found in 14,546 publications from 1978 onwards. Finally, the search query ‘hematopoietic stem cell niche’ determines more than 4000 papers since Schofield’s paper in 1978; 62% of these (2564 papers) were published in the years 2012–2021. It is noteworthy that, according to the PubMed search engine (https://pubmed.ncbi.nlm.nih.gov/; accessed on 7 May 2025), the maximum number of annual publications in this field was reached in 2021 (342 references). By 2024, the number of published papers decreased to 208.

Similarly, the word combination ‘MSC niche’ showed a total of 1037 results in the PubMed database from 2004 onwards. The peak (105 publications) was observed in 2020, with the number of articles published annually decreasing to 65 (by 38%) by 2024. Thus, although several more or less comprehensive reviews and experimental studies have been published in recent years demonstrating the progress in stem cell niche research, certain stagnation can be observed in this field, possibly due to different opinions, broad interpretations, and a lack of solutions to the fundamental and applied issues of the “niche” concept. In particular, there is still no answer to the key question: how do the components of a niche as a specialized microterritory differ from the same well-known components of the entire HIM?

In this review, we discuss recent findings about the major milestones of the niche concept, focusing on BM-derived HSCs and MSCs as the most extensively studied yet more complex systems as we have understood them so far [13,14], and highlight the problematic questions that remain to be explored at the threshold of 50 years of the stem cell niche concept. We propose that these problems can be partially overcome by establishing a consensus among experts in regenerative medicine, developmental biology, and bioengineering using the niche questionnaire we have developed (Table S1).

The term “niche” was used by Calvo et al. (1976) to describe the osteal sites in the trabecular bones where groups of cells (colony-forming units, CFU) demonstrated either erythropoiesis, myelopoiesis, or megakaryocytopoiesis during a recovery after lethal irradiation [15]. In other words, distinct BM microenvironments have been identified in which cellular components contribute to the niche, many of which regulate differentiation scenarios.

In 1978, Schofield proposed a theoretical basis in the form of a “niche” hypothesized as a specialized site of HIM to maintain the HSC self-renewal and stemness [9,16].

Despite the obvious progress of Schofield’s hypothesis, the spatial organization of BM hematopoiesis remains poorly understood. Hence, since the birth of the term “stem cell niche”, there is no certainty in its interpretation so far. The understanding of the niche for cells of mesenchymal origin by different authors contradicts each other in key aspects. These opinions can be divided into two large directions: the orthodox variant versus the alternative point of view (Table 1 [13,14,15,16,17,18,19,20,21,22,23,24,25,26,27,28,29,30,31,32,33,34,35,36,37,38,39,40,41,42,43,44,45,46,47,48,49,50,51,52,53,54,55,56,57,58,59]).

Some orthodox variants of niche definition are as follows:

1. The fundamental property of a stem cell is self-renewal, which depends on the microenvironment in which the stem cell is seen in association with other cells, determining its behavior [10].
2. The niche is a highly specialized, complex, and instructive microenvironment that physically localizes the stem cells and maintains their fate [17,18].
3. The stem cell niche is a specialized microenvironment in which stem cells reside primarily in a quiescent state by providing anti-proliferation signals [19].
4. The stem cell niche is a specific microenvironment that directly controls the dual capacity for self-renewal and multilineage differentiation of stem cells, maintaining the HSC in a quiescent state [20].
5. The niche is a restricted site in an organ that supports the stem cell self-renewal [21].
6. Stem cells reside in specialized microenvironments, also called niches, which maintain them in an undifferentiated and self-renewing state [22].
7. Niches are the anatomical regions within the microenvironment [60].

Forty years after Schofield’s article, Purton and Scadden (2008) have formulated several questions that are largely fundamental to the development of the HSC niche hypothesis, particularly: are all niches equal; do one or numerous niches exist; are the niches in a quiescent or activated state; and does niche hierarchy correspond to stem cell hierarchy [16]? As a continuation of these questions, Lucas (2021) considers distinct niches for HSCs and their progenitors in the BM [61]. Based on new proposals and accumulated experimental data, alternative definitions of the HSC niche have emerged, some of which we present below.

An alternative point of view reflecting the hematopoiesis dynamicity is presented by certain descriptions:

1. The stem cell niche is a group of cells in a special tissue location that are intended for the maintenance of stem cells. It has a variable structure, and different cell types can provide the niche environment. There is a niche hierarchy of HSC due to HIM heterogeneity at diverse regions of the BM [23].
2. There are the hierarchical HSC osteoblastic (and MSC) niches in terms of their localization, composition, function, and multi-layer regulation systems [24].
3. The stem cell niches are the distinct, structurally functional, energetically favorable microterritories in a contiguous space of HIM where quantitative parameters of a microenvironment promote the qualitative control of stem cell fate [13].
4. According to Pinho et al. (2018), the HSC pool is functionally and molecularly heterogeneous; consequently, there are distinct ‘specialized’ niches for distinct subpopulations of HSCs [25].
5. The HSC niche is considered to be a complex multicellular network that provides molecular signals and physical interactions that are essential for HSC localization, maintenance, and differentiation [26].
6. The niche provides a microenvironment that supports the self-renewal and multi-lineage differentiation of stem cells [27].
7. The niche represents a sophisticated and dynamic system of cellular and molecular components coupled with heterogeneous signaling mechanisms. It serves as an interface between stem cells and the organism, orchestrating their adaptive responses to tissue damage [62].
8. The BM niche is a complex environment composed of heterogeneous cell populations that regulate the hematopoietic stem and progenitor cells (HSPCs) function and activity through the secretion of a wide array of cytokines and growth factors [63].
9. The stem cell niche is a dynamic and specialized microenvironment with a specific architecture that regulates self-renewal of stem cells, the balance between their quiescent and proliferative status, as well as their choice of fate and differentiation of their progenitor cells [28].

The mentioned definitions could also be applied to the niches of other tissue-specific stem cells. However, it should be noted that differences in structure and functional features are observed for distinct types of stem cell niches belonging to epithelial or stromal niches (reviewed in detail in [62]).

Briefly, epithelial niches (such as niches for intestinal stem cells, epithelial stem cells, hair follicle stem cells, limbal stem cells, spermatogonial stem cells, etc.) are generally determined as a specific microterritory in the tissue, and their components are usually clearly distinguishable by histological analysis. On the contrary, stromal niches (HSPC and MSC niches, etc.) have an elusive microanatomical structure and are characterized by a pronounced dynamicity. Below, we will discuss the key components of all stem cell niches and the critical regulatory mechanisms involved in the interactions between a stem cell and its niche, focusing mainly on BM niches.

### 2.1. Core Components and Regulatory Mechanisms in Stem Cell Niches

#### 2.1.1. Cellular and Non-Cellular Components of Stem Cell Niches in Mammalian Tissues

**Figure 1:**
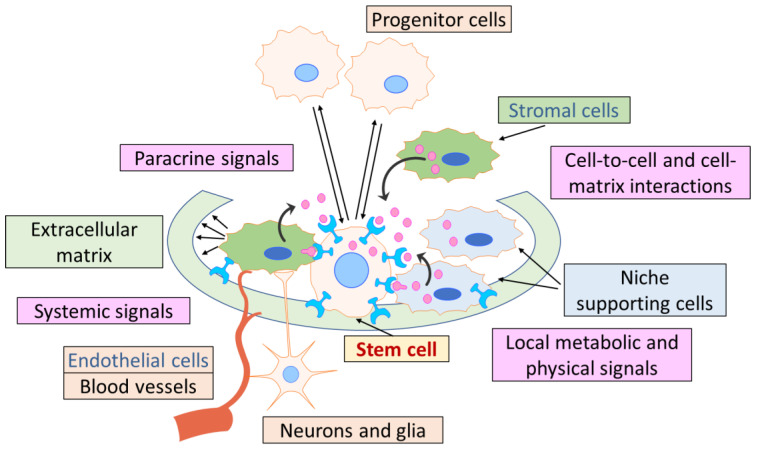
Key components of a stem cell niche. The stem cell niche provides a structural and regulative microenvironment for stem cells, supporting their maintenance and adequate response to the differentiation stimuli. The existing opinion is schematically emphasized: a niche is a small part of the microenvironment, which nevertheless has the same structural and functional organization as the broader HIM space. Please see the text for additional details.

The stem cell niche in postnatal tissues, being a sophisticated, heterotypic, and dynamically regulated microenvironment, was shown to be composed of multiple interacting components of different natures (Figure 1). The cellular components consist of adjacent niche-supporting cells, MSCs, immune cells, and parenchymal cells; the noncellular components include the extracellular matrix (ECM), signaling molecules (e.g., growth factors and cytokines, extracellular vesicles, etc.), physical cues (e.g., shear stress, substrate rigidity, and topographical features), and extrinsic environmental stimuli (e.g., glucose and metabolic byproducts, oxygen tension, small ions, and inflammatory mediators) (Figure 1). Importantly, stem cell niches require extensive vascularization and neural integration, making them susceptible to both direct and indirect modulation by circulatory and neuronal inputs as systemic signals [62,64,65,66,67,68].

The stem cell niche comprises multiple cell types from distinct lineages that collectively regulate stem cell maintenance and their response to activating stimuli. Thus, in the HSC niche within the BM, stromal cells such as osteoblasts and MSCs, endothelial and immune cells, together with distinctive signaling factors, regulate the balance between HSC quiescence, activation, proliferation, and differentiation to maintain the blood cell system. HSCs reside in osteoblastic niches near the endosteal bone surface and in perivascular niches (including periarterial, perisinusoidal, and perivascular in the transition zone), where they communicate locally with their cellular microenvironment through cell–cell interactions and secreted factors or extracellular vesicles released into the BM [69]. The osteoblastic niche may provide a quiescent microenvironment for HSC maintenance, whereas the vascular niche facilitates HSC transendothelial migration during mobilization or homing and may favor HSC proliferation and further differentiation. Quiescent HSCs are adjacent to the endosteal lining, where they are in close contact with N-cadherin+ osteoblasts, which regulate HSC quiescence through secretion of osteopontin and angiopoietin. Activated HSCs are attracted to the central marrow region in the perivascular niche, close to the sinusoid, where they can give rise to multipotent HSPCs and subsequently new blood cells, which are then released into the circulation [70]. Leptin receptor-expressing (LepR+) perivascular MSCs and endothelial cells that surround sinusoids and potentially other blood vessels throughout the BM are required for HSC maintenance [71]. It was found that CD150^+^CD48^+^CD41^−^ lineage-negative HSCs localize in the perivascular zone near sinusoidal blood vessels in close contact with not only vascular and perivascular cells but also megakaryocytes and other hematopoietic cells [30].

Recent advances in biomedicine have established the crucial role of MSCs in the maintenance of many stem cell niches in various tissues [12,72,73,74]. One of the most studied niches in this context is the niche of HSCs. In the hematopoietic niche, separate subpopulations of MSCs have been described, and they are closely connected with HSCs due to intercellular contacts and also produce various factors supporting hematopoiesis [73,74]. Deletion of the population of MSCs expressing the marker nestin resulted in impaired homing of HSCs to the BM in transplantation after complete irradiation of the recipient BM. In addition, MSCs are known to support hematopoiesis ex vivo when HSCs are isolated in culture and can also form an HSC niche-like microenvironment in heterotopic transplantation in vivo [75]. Human skeletal stem cells (SSCs), often referred to as MSCs in skeletal tissues, were shown to have a capacity to maintain human HSCs in serum-free culture conditions ex vivo [76].

Accumulating evidence demonstrates that the specific population of MSCs, termed CXC chemokine ligand 12 (CXCL12)-abundant reticular cells (CAR), which overlap with LepR+ cells, represents the crucial cellular component within the perivascular HSC niche [68]. CAR cells, as well as endothelial and perivascular stromal cells, produce CXCL12 and stem cell factor (SCF), regulating HSC maintenance and activation. Macrophages in close proximity to nestin-expressing MSCs can modulate the secretion of CXCL12 by MSCs [77].

MSCs could also support the multipotent stem cell subpopulation within their heterogeneous population, which is involved in the stromal tissue renewal and regeneration. Thus, ECM components produced by MSCs could promote the differentiation of multipotent stem cells into the adipogenic, osteogenic, and chondrogenic lineages [78].

In the epithelial stem cell niche, i.e., the intestinal stem cell niche, the population of Gli1-positive MSCs, located in the depth of the intestinal crypt, is one of the key participants of niche regulation through the production of ligands of the Wnt signaling pathway, even when the function of the main sources of these factors—Paneth cells—is impaired. MSCs also negatively regulate intestinal stem cells via bone morphogenetic protein 2 (BMP2) and BMP4 secretion [77]. In the hair follicle niche, MSCs participate in the activation of stem cells by secreting Noggin and Wnt factors as well as Shh [79]. In the spermatogonial niche, MSCs regulate the functioning of Leydig cells and Sertoli cells, thus stimulating the restoration of the niche after damage [80]. MSCs may also be involved in the regulation of stem cell niches in other tissues. However, there is little experimental data on this, and the specific mechanisms by which MSCs exert their regulatory function in the niche remain poorly understood.

Other cell types, such as endothelial and immune cells, have recently attracted increasing attention from researchers as regular contributors to stem cell regulation within a niche. Thus, in the BM, endothelial cells interact with osteolineage cells in a paracrine manner (so-called angiogenic-osteogenic coupling) and, together with perivascular stromal cells, also directly form the perivascular niche for HSCs. Recent studies highlight the heterogeneity of vascular niches at different bone regions, suggesting that HSCs are regulated by locally distinct mechanisms [81]. Immune cells, classical hematopoietic components derived from HSPCs, such as neutrophils, macrophages, dendritic cells, B cells, and others, mature predominantly in the BM and are considered important cellular regulators of HSC quiescence, self-renewal, and multi-lineage differentiation through either direct cell-to-cell interactions or the secretion of various immunological factors. They regulate not only HSCs but also other cells within the niche, such as MSCs, endothelial cells, osteoblasts, and osteoclasts, to promote their survival and modulate the supportive microenvironment for HSPCs [82].

Adipocytes derived from CAR/LepR+ cells could act as negative or positive regulators of HSCs during functional hematopoiesis and the myelosuppressive condition (discussed in detail in [68]). Within the complex BM microenvironment, HSCs constantly cooperate with the SSC niche to maintain homeostasis and regulate skeletal formation as well as immune system functioning [83]. There is also a constant neural-immune crosstalk regulating niche activity by the contact with sympathetic neurons [84].

Recent advances in the development of novel genetic and histology tools, including single-cell RNA sequencing and spatial multi-omics, have made it possible to elucidate cellular heterogeneity and specific cell populations within the HSC niche and other stem cell niches and generate single-cell atlases to decipher the microenvironment of stem cells [69]. These approaches provide novel data regarding the cellular components of stem cell niches in BM and other tissues, expanding our understanding of how the niche is regulated.

Among the non-cellular components of the niches involved in the regulation of stem cell functioning, the ECM is considered to be one of the most important. The ECM represents a dynamic and intricate microenvironment distinguished by tissue-specific biophysical, mechanical, and biochemical properties that play a crucial role in modulating stem cell behavior [74]. The multiple components of the ECM exert direct and indirect control over stem cell maintenance, proliferation, self-renewal, and differentiation [85]. Various ECM molecules exhibit regulatory functions across different stem cell types, and the ECM’s molecular composition can be precisely assembled and adjusted to establish an optimal niche for stem cells in specific tissues (reviewed in detail in [36]). Integrins, expressed on the surface of stem cells, are often involved in the interaction with ECM components within a niche. Thus, it was demonstrated that the αv integrin subunit could regulate HSC proliferation via interaction with periostin [86].

Intercellular communication within stem cell niches occurs through multiple modalities, including direct cell–cell contact, secreted factors, and extracellular vesicles, which enable both local and long-range coordination of cellular activities [74]. Cell adhesion molecules play particularly important roles in maintaining proper spatial organization and facilitating direct signaling between adjacent cells. Among the signaling pathways, Wnt, Notch, BMP, and transforming growth factor (TGFb) can be highlighted as critical regulators of stem cell self-renewal and cell fate. These evolutionarily conserved pathways enable precise control over cell fate decisions while maintaining the flexibility required for appropriate responses to physiologic demands. Extracellular vesicle-mediated communication also represents an emerging area of research that may provide new insights into how niche cells coordinate their activities over longer distances.

The molecular regulation of stem cell niches relies heavily on sophisticated chemokine and cytokine signaling networks that coordinate cellular behavior and maintain tissue homeostasis. CXCL12 and SCF represent two of the most critical regulatory molecules within the BM niche and serve as essential regulators that manage HSC behavior, including quiescence, proliferation, migration, and differentiation [74]. SCF binds to c-Kit on HSCs and is essential for their repopulating activity. It exists in both membrane-bound (mSCF) and soluble (sSCF) forms, with the former being more potent in retaining HSCs within their niche [69].

CXCL12, also known as stromal cell-derived factor 1 (SDF-1), is a chemokine that plays a crucial role in stem cell niches. In the BM, it is essential for the survival, maintenance, and trafficking of HSCs [87]. CXCL12 acts through its receptor CXCR4, which is expressed on HSCs, to promote their quiescence and self-renewal. In response to changes in CXCL12 levels, a portion of HSC progenies leave the niche and begin to mobilize and circulate. Targeted deletion of CXCL12 from MSCs in the BM was shown to reduce the normal HSC numbers [88].

The process of HSPC recruitment into the vascular niche may also depend on endothelium-derived fibroblast growth factor 4 (FGF-4) and differences in oxygen level. Higher FGF-4 and oxygen concentration gradients during the transitions of cells from the osteoblastic niche to the vascular niche regulate the recruitment, proliferation, and differentiation of HSPCs. Under stress such as thrombocytopenia, SDF-1 and vascular endothelial growth factor (VEGF) activate metalloproteinases (MMP) like MMP-9, which convert membrane-associated Kit ligand to soluble Kit ligand (sKitL), thus promoting HSC entry into the cell cycle, mobilization to the vascular niche, and differentiation [47].

While most studies of the niche focus on those residing in the BM, HSC niches in extramedullary tissues are also actively studied. Thus, HSCs were found to reside in adult mouse meninges [89]. Locally expressed CXCL12 and SCF maintain meningeal HSCs and regulate their adaptation to the central nervous system immunosurveillance. These cells could be replenished from blood upon niche depletion. Gao et al. demonstrated that epitranscriptomic control resulting in RNA modifications (e.g., m6A) dictates niche-specific HSC fates [90]. Tissue-specific HSC niche was also described in spleen [91], where it functions under the megakaryocyte-driven regulation and neural signals, adipose tissue and lungs [92], as well as placenta and fetal liver [93]. However, extramedullary HSC niches, especially in human tissues, remain understudied.

In other stem cell niches, the mediators of intercellular communication may vary depending on the rate of cell turnover and the specificity of niche-supporting cells, but the crucial principles of the regulation are similar to those in the HSC niche—a balance between stem cell maintenance and activation on demand. Thus, in the intestinal crypt Leucine-rich repeat-containing G protein coupled receptor 5 (LGR5)+ intestinal stem cells are located at the base of the crypt, embedded between their specialized daughter cells, the so-called Paneth cells, which supply Wnt, epidermal growth factor (EGF), Delta-like protein 4 (DLL4) and Noggin ligands necessary for the self-renewal and maintenance of stem cells [77]. In the spermatogonial stem cell niche, the population of stem cells strongly depends on the supply of fibroblast growth factors (FGFs) secreted by lymphatic endothelial cells, which provide the competitive microenvironment regulating stem cell homeostasis under the conditions of limited mitogen availability [94,95].

Considering the high complexity and heterotypic structure of the stem cell niches, it is still controversial which components and mechanisms lead to the regulation of stem cells within their niches. These questions are included in the questionnaire developed to consolidate expert opinions on this issue (Table S1).

#### 2.1.2. Comparison of HSPC Niche Structure and HIM Morphologic Components in the BM

This morphofunctional section of the niche concept can be regarded as the most advanced since the formulation of Schofield’s hypothesis and is constantly evolving. This is not surprising, since the niches are the specialized HIM territories (surrounding the HSPCs); the HIM concept has been evolving since the 1960s [6,96].

At the present time, the intravital microscopy technique allows topographical visualization in the BM and in situ detection of the function of numerous cell types, including bone cells, megakaryocytes, macrophages, perivascular cells, and Schwann cells, as niche regulators of HSC quiescence, mobilization, and responsibility [26,52]. Mature hematopoietic and innate immune cells (macrophages, dendritic cells, B cells, Breg cells, CD4^+^ T cells, CD8^+^ T cells, Treg cells, and neutrophils) are proposed to be the regulators of the HSPC niches through feedback mechanisms [82,97].

HIM morphologic components were previously defined (prior to Scofield’s publication) as follows [98]:

1. Tissue-resident and migrating cells.
2. ECM with fibers and soluble molecules.
3. Blood vessels.
4. Nerve terminals.

The main components of the HSPC niche, in turn, are described in multiple publications (e.g., [13,26,29,30,31,32,33,34,35,36,99]) and consist of the following:

1. Various hematopoietic and stromal support cells, including cell–cell adhesion molecules and secreted soluble factors, are located in close proximity to stem cells.
2. ECM, which serves as an “anchor” for stem cells and forms a mechanical scaffold for the transmission of stem cell signaling.
3. Blood vessels that supply the niche with nutrients and systemic signals from other organs and are involved in the recruitment of circulating stem cells from and into the niche.
4. Nerve terminals and Schwann cells.

Comparing the core components of the HIM space and the HSPC niches, their strong similarity can be easily noticed. Onward, briefly examining general regulatory mechanisms, Tavassoli (1975) has described that HIM components control the HSC fate via direct (cell–cell and cell–matrix) and indirect (soluble molecules) cues [98]. The functioning of the stem cell niche, in turn, is based on both the physical contacts and diffusible factors (e.g., [26,100]). It appears that the control mechanisms of HSPC behavior in the niche and in HIM are largely similar.

Of course, significant progress has been made recently in deciphering the intimate processes of both cellular components (MSCs, inflammatory and immune cells, megakaryocytes, etc.) [75,97,101,102] and molecular signaling (exosomes, integrins and other anchor molecules, chemokines, hypoxia, etc.) [103,104,105,106,107] within the hematopoietic microenvironment.

However, virtually all cells, molecules, and cues in the BM can be considered to be components of one or another stem and/or progenitor cell niche (Table 1). This gives rise to the following controversial assumptions:

1. The entire HIM space of the BM consists of niches for HSCs, MSCs, and their progenitors, which are in close contact with each other.
2. Between the niches, there is a non-specialized HIM space, which has extrinsic effects on its functioning.
3. It is unclear whether there are significant (qualitative or quantitative) differences between the local specialized microterritories (referred to as the niches) and the known components of the entire HIM space. What are the external borders of stem cell niches to distinguish their structural and functional features?

These and other crucial issues, also summarized in Table 1, have largely shaped the further argumentation and conclusions in the present review. As it is crucial to understand the mechanisms of stem cell niche regulation, we dedicated the selected part of the developed questionnaire to the issues related to the control of stem cells within a niche (Table S1).

## 3. Hierarchy of Potential Niche-Candidates for Stem and Progenitor Cells

The niche supporting HSPCs in the BM is a highly heterogeneous and dynamic structure that responds to changing physiologic or pathologic signals [52,97]. Therefore, Yu and Scadden (2016) [52] viewed the BM environment (HIM; [6]) as composed of multiple micro-niches, each consisting of a unique pairing of distinct supportive stromal cells with different hematopoietic subtypes to regulate a particular branch of hematopoietic cell process.

**Figure 2:**
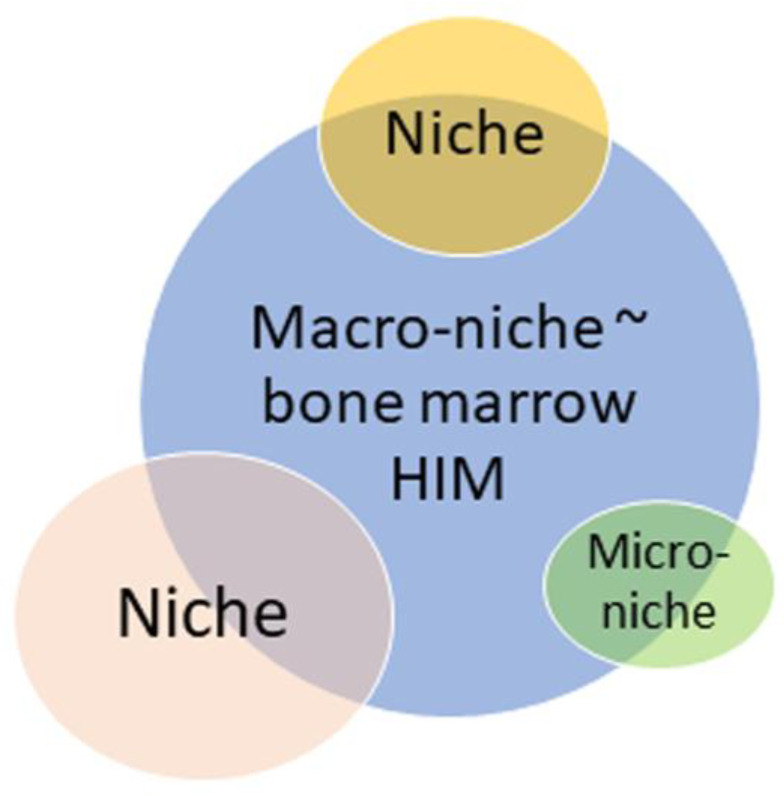
Bubble map of understanding size variability for distinct HSC candidate niches. HIM—hematopoietic-inductive microenvironment. The areas of bubbles reflect schematically the variations in the unitless value of the niches described by different authors. The figure begs the question about certain borderlines and dimensions of the niche; otherwise, it can be considered ‘cloud’ (functional) subtypes only.

In particular, Baccin et al. (2020) described perivascular micro-niches established by CAR cell subsets (Adipo-CAR and Osteo-CAR) that localize differently on sinusoidal and arteriolar surfaces [53]. Conversely, Kandarakov et al. (2022) considered mammalian BM as a macro-niche for HSCs [40]. These hypothetical speculations about Schofield’s niche, as well as micro- and macro-niches, blur the lines of niches from single cell associations to the entire hematopoietic organ. In this case, the term ‘niche’ equated with the term HIM (Figure 2).

Since the size and composition of the HSC microterritories are still unclear, even the classical Schofield’s niche can vary significantly in value (Figure 2). For example, macrophages and megakaryocytes vary significantly in their dimensions; thereafter, megakaryocytic candidate niche [44] and erythroblastic islands [42] appear to be somewhat different BM microterritories.

Regarding BM as a macro-niche, Kokkaliaris et al. (2020) [108] have shown interesting results. It was found that the distribution of HSCs relative to the candidate niches was stochastic and did not differ from a mapping of computationally generated points randomly placed throughout the entire BM volume. Finally, Kandarakov et al. (2022) [40] supported the idea of [52] that HSC niches are anatomically abundant and are not a limiting factor in vivo.

**Figure 3:**
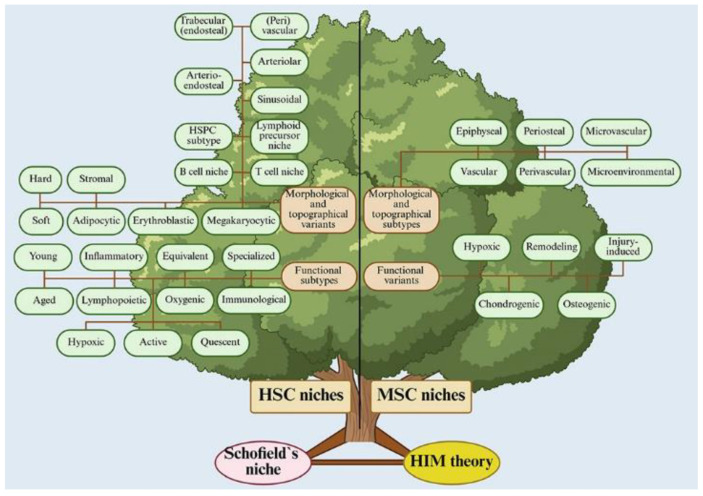
Candidate niche tree for bone marrow HSCs and MSCs described in the references. Drawn from data presented in Table S2 with corresponded references [11,13,26,28,40,41,42,43,44,45,46,47,48,49,50,52,54,56,61,110,111,112,113,114,115,116,117,118,119,120,121,122,123,124,125,126,127,128,129,130,131,132,133,134,135,136].

In the current literature, various authors have described a wide range of cells and structural-functional units that are assumed to be candidate niches for HSPCs, as well as for stromal cells themselves, including MSCs. Since our previous review [54], the list of candidates for HSPC and MSC niches has expanded considerably. It can be conditionally divided into structural-functional, topographical, and functional variants (Figure 3, Table S2).

Of particular interest among candidate HSC niches are hematopoietic (erythroblastic) islands and megakaryocytes as native BM cells and structural-functional units, whose size is easy to determine. It can serve as a dimension scale for separating other cell associations that are not always correctly considered as stem cell niches [109].

### 3.1. Erythroblastic Islands as Candidate Niche for HSPCs

Almost 70 years ago, the erythroblastic islands (EIs) were primarily defined by Bessis as structural-functional units of the BM [116]. The EIs have been currently updated to be renamed as ‘erythroblastic island niche’ [117,118,137], supported by a subset of macrophages that provide erythroblast maturation near sinusoid vessels [117].

**Figure 4:**
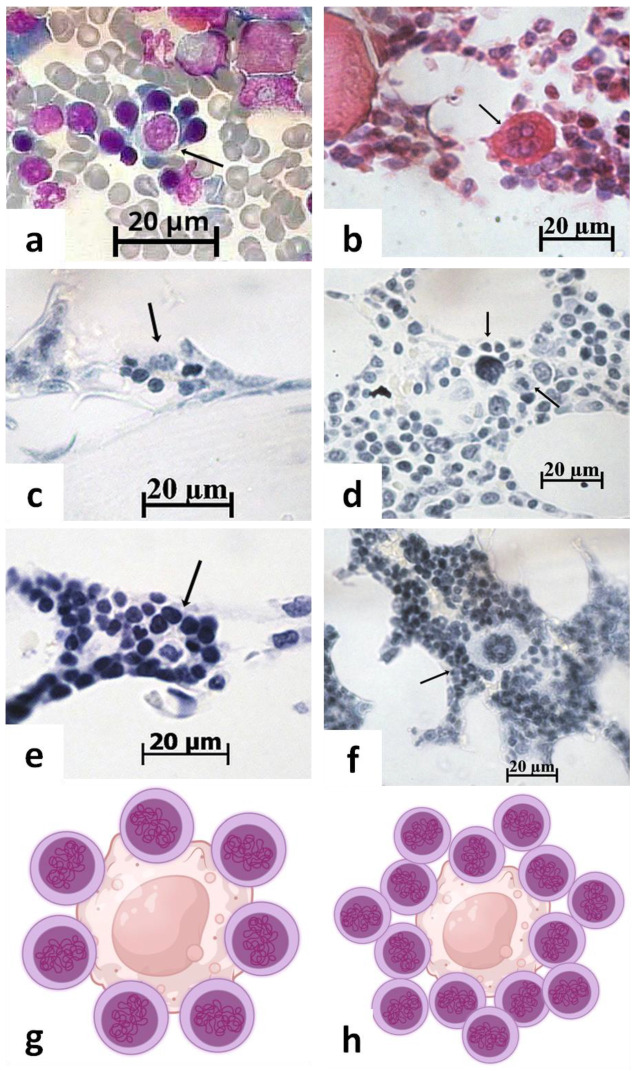
Composition of hematopoietic islands (**a**,**c**,**e**) and megakaryocytic niche-candidate (**b**,**d**,**f**) on cytological smear (**a**) and histological sections (**b**–**f**) of mouse (**a**), rabbit (**b**–**d**,**f**), and human (**e**) bone marrow. Black arrows specify cell associations. (**a**) Erythroblastic island. Azure II and eosin staining; (**b**) megakaryocyte rounded by hematopoietic cells. Hematoxylin and eosin staining; (**c**,**e**) hematopoietic islands with different nucleic acid (DNA and RNA) contents in hematopoietic cells; (**d**,**f**) megakaryocyte surrounded by single (**d**) or multicellular layers (**f**). The Einarson staining; dividing cell (black arrow) near megakaryocyte (**d**). Scale bar 20 µm; (**g**,**h**) schematic 2D reconstruction of hematopoietic niche-candidate: central stromal cell (macrophage, fibroblast-like cell, or megakaryocyte) surrounded by single (**a**,**c**,**d**) or multilayered (**b**,**e**,**f**) hematopoietic cells. Own microscopic images and cartoons are shown.

Ex vivo EI reconstitution is used to mimic an interaction of HSCs with macrophages in the natural BM environment under different stress conditions [138]. In all this, Han et al. (2023) [138] consider the unclear relation of EIs reconstructed in vitro to their in vivo features. Hematopoietic islands (HIs) can be easily identified in medullary tissue by standard cytologic [56] and histologic techniques (Figure 4). HIs consist of immature hematologic cells that actively synthesize the nucleic acids (Figure 4c,e) and incorporate ^3^H-thymidine [56]. It is proposed to consider HIs as active hematologic niches.

Moreover, HIs have an apparent size (Figure 4a,c,e) that may be useful for the comparative evaluation with the dimensions of other niche candidates. In particular, such an estimation was recently conducted by [109].

Interestingly, Sathyanarayana et al. (2007) hypothesized the existence of a stromal niche for early-stage erythropoiesis [139], while EIs consisting of about 5–30 erythroid cells surrounding a central resident macrophage have been proposed as niches for the proliferation, differentiation, and enucleation of late-stage erythroid precursors [42,117,140].

Indeed, the central stromal elements within the EIs are surrounded by erythroid cells (Figure 4a), with varying concentrations of nucleic acids in their nucleus and cytoplasm (Figure 4c,e), stained using the well-known Einarson technique [141].

### 3.2. Megakaryocytic Candidate for HSPC Niches

Bruns et al. (2014) showed that about 30% of HSCs are localized near megakaryocytes (MKs), which promote stem cell quiescence via CXCL4 production [44]. Later, Pinho et al. (2018) found ~60% of von Willebrand factor-positive HSCs within 5 μm distance of MKs [25]. In summary, they suggested that MKs maintain HSC quiescence and may be specialized hematopoietic niches [26,118].

However, there are serious contradictions that MKs are primarily the niches and secondarily promote the HSC quiescent state. Thus, Lucas (2021) proposed the existence of a megakaryocyte/sinusoidal niche that maintains HSCs biased towards megakaryocyte fates and an arteriolar niche that maintains more quiescent HSCs [61].

In turn, Kandarakov et al. (2022) suggested the possibility of direct contact between MKs and HSCs due to the cell–cell interaction through chemokines (CXCL4) and growth factors (TGF-β1; thrombopoietin; FGF1) secreted by MKs [40]. If the authors’ hypothesis is correct, MKs are mainly part of the mutual HIM but not a specialized microterritory for the HSCs. Moreover, light microscopy of BM histology clearly confirms a close contact of MKs with hematopoietic cells (Figure 4b), which actively synthesize DNA and RNA molecules (Figure 4d,f). There are some cells with a mitotic figure (Figure 4d, black arrow). Therefore, hematopoietic quiescence near MKs has not been confirmed morphologically, and the role of MKs as potential niches for HSCs remains controversial.

### 3.3. MSC Niche

SSCs play a crucial role in shaping the medullary cavity by providing both the structural framework and the hematopoietic microenvironment necessary for BM function [142]. These cells establish specialized niches, including SSC-derived HSC microenvironments tailored to specific physiologic needs [76,143].

However, SSCs exhibit significant heterogeneity, with distinct populations residing in articular cartilage, the growth plate, periosteum, and perivascular regions. Remarkable progress has been made in characterizing various SSCs and skeletal stem and progenitor cell (SSPC) populations in postnatal bone. Their isolation, detection, and functional characterization remain debatable, which is exacerbated by inconsistencies in nomenclature—such as the interchangeable use of “SSCs” and “mesenchymal stem cells (MSCs)” [143]. Consequently, the existence of SSPC niches in the BM remains under discussion [144]. SSPCs were localized in the periosteum and within the BM stroma, including subsets localizing around arteriolar and sinusoidal blood vessels; they can display osteogenic, chondrogenic, adipogenic, and/or fibroblastic potential, and exert critical hematopoiesis-supportive functions (for a detailed review, see [145]).

Despite these uncertainties, recent studies employing advanced in situ techniques—including laser-capture microdissection, confocal fluorescence microscopy, and immunohistochemistry—suggest the presence of an SSPC niche in the postnatal epiphyseal growth plate in mice. These niches appear to support sustained chondrogenesis over prolonged periods [50]. Other potential SSPC niches have been proposed in other anatomical regions, such as articular cartilage and periosteum [28,146].

The interpretation of these findings is complicated by conflicting data. For instance, a combination of micro-CT imaging, molecular profiling, and clonal cell tracing failed to identify stem cell niches in salamander cartilage during development or regeneration [147]. Nevertheless, many studies highlight the spatial association of MSC niches with bone, cartilage, and the microvasculature (Figure 3, Table S2), as pericytes—often considered as MSCs—can differentiate into osteoblasts, chondrocytes, and adipocytes [148]. The precise regulatory role of these niches in determining MSC fate, HSC behavior, or both remains unclear. However, emerging evidence suggests the existence of functionally distinct MSC niches (e.g., osteogenic, chondrogenic) (Figure 3, Table S2).

Despite these challenges, ex vivo modeling offers a promising approach to predict the structural and dimensional characteristics of functional MSC niches, facilitating their identification in native bone [43,54]. In combination with in vivo imaging, such models could provide critical insights into the quantitative and spatial organization of the stem cell microenvironments across different tissues.

### 3.4. Live Imaging of Niches

Intravital microscopy is a rapidly developing field for niche detection within the mammalian BM [149]. For example, many studies have attempted to determine which of the two cellular elements in the BM, osteoblasts or endothelial cells, could serve as a niche for long-term HSCs [30]. In this context, pioneering live imaging work that has captured labeled HSCs within their normal 3-dimensional tissue environment has helped to address this issue by suggesting that HSC niches may indeed contain both osteoblast and vascular components that are closely apposed [150]. Multiphoton laser microscopy (MLM) can penetrate the outer bone thickness (~50 µm in the murine cranium) and then image in the marrow region at an additional depth of 250 to 300 µm [151] with high (subcellular) resolution (0.5 µm in the lateral direction and 1–2 µm in the axial direction) [152].

At the same time, MLM is unable to penetrate into the deepest regions of the BM; therefore, it is complicated to observe the niches for short-lived HSCs and multipotent progenitors maintaining day-to-day hematopoiesis in adult animals [153]. In addition, according to Pinho et al. (2019), the location of a stem cell within tissue is not sufficient to define its niche, as the niche must have both anatomical and functional features [26]. In particular, determining the site of a labeled stem cell in the BM does not allow us to outline the borders of its specialized maintaining area in the general space of the HIM. This can only be assumed, since the currently known cellular and molecular components of the niche and the entire HIM are, on a large scale, the same.

### 3.5. Niche Aging as One of the Functional Manifestations of Specialized Microterritories

The stem cell niche is the most important intrinsic factor regulating cellular aging in adult stem cells [20]. In particular, Pinho et al. (2019) consider aging-related changes in the BM niche that influence HSC aging as the alterations in the vasculature and mesenchymal stem and progenitor cells, increased adipogenesis and reduced osteogenesis, altered secretion of niche factors, and a reduced number of adrenergic nerves [26]. According to a review by Kandarakov et al. (2022), aging-related alterations occur in HSCs and niche-supporting cells (MSCs, endothelial cells, osteoblasts, adipocytes, MKs, macrophages, and lymphocytes) and components (nerve fibers, molecule secretion) due to the increased proinflammatory molecules and cells [40].

Thus, Young et al. (2024) showed a reduction in cues from MSCs to HSCs in old mice, resulting in HSC impaired lymphoid differentiation in aged niches versus balanced lymphoid/myeloid signaling in young HSC microterritories [45]. Aged HSCs exhibit high proliferation with increased myeloid-biased differentiation and reduced regenerative capacity, which promotes myeloid malignancies [26].

In addition, neutrophils, as contributors to the HSPC niche [97] and proinflammatory cells, may support hematopoietic niche aging. Therefore, Yang and de Haan (2021) [154] highlighted the emerging role of inflammation in HSC aging, leading to low functionality of aged HSCs, including a declined repopulation capacity and myeloid and platelet-restricted differentiation. Taken together, both cell-intrinsic and microenvironmental extrinsic cues contribute to HSC aging. 

## 4. Challenges of the Dimension and Geometry of Stem and Progenitor Cell Niches

Lovegrove et al. (2025) [155] have recently shown an instructive influence of pre-mitotic cell morphology and shape (spherical or spindle-like) on its symmetric (via mitotic rounding) or asymmetric (isomorphic) division. The isomorphic mode triggered heterogeneous morphology, unequal distribution of signaling pathways, determinant polarization, and, finally, variable differentiation of mesenchymal-derived daughter cells. Accordingly, the dimensions and geometric features of specialized HIM sites (niches) can also influence the morphogenetic events in stem cells due to the precise control of gene expression during organogenesis [156].

The specific niches for the distinct developing hematopoietic lineages are poorly understood; still less is known about the changes in niche size and function in these distinct BM anatomical sites under stress conditions and aging [157]. Niche features are not only important for the control of malignancy processes of stem and precursor cells [17,21,158]. The bioengineering phase of the niche concept development [13] requires precise characteristics of the stem cell microenvironment to mimic biological niches by designing artificial microterritories that have structural, functional, and biomechanical parameters similar to the natural conditions. However, there are some crucial quantitative properties of niches [54] that need to be replicated ex vivo. The lack of consensus on the generally accepted dimensions and geometry of the specific microenvironment for stem cells makes their biomimetic reconstruction, for example, using modern additive technologies, for the needs of regenerative medicine, impossible.

**Figure 5:**
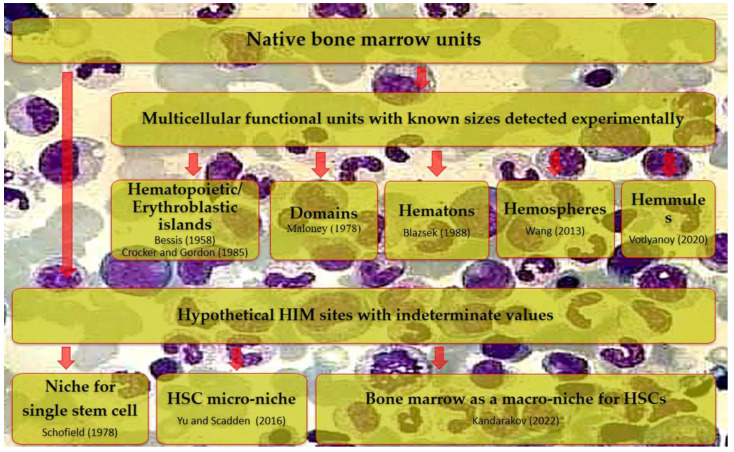
Some native sites of the BM hematopoietic inductive microenvironment (HIM) have been found or proposed in vivo. BM—bone marrow, HSCs—hematopoietic stem cells. The figure highlights the authors’ view about the necessity for transition from theoretical discussions about the existence of hypothetical stem cell niches to the definition and expert approval of their composition, dimension, and bounds, as has been described for larger cell associations detected in the BM. Following references are cited here: [9] Schofield (1978); [24] Wang (2013); [40] Kandarakov (2022); [52] Yu and Scadden (2016); [55] Maloney (1978); [56] Crocker and Gordon (1985); [57] Blazsek (1988); [59] Vodyanoy (2020); and [116] Bessis (1958).

Recently, we have attempted to compile the available quantitative information on the natural microterritories for mesenchymal cells (HSCs, HSPCs, and MSCs) [109]. It turned out that the size of niches as associations of a few cells is poorly represented in the available literature. Much more is known about the scales of other, larger cell associations in the BM (in particular, “domains”, Figure 5), which are often referred to as “niches” in compliance with the existing trend.

BM domains were described by Maloney et al. (1978) in the same year as Schofield’s niche [55]. The domain represents a large (up to 0.1 mm^3^; cube of 50 cell diameters) multicellular association (~90,000 cells) of hematopoietic and stromal elements. The results were further developed experimentally by other authors, establishing various cellular formations (hematons, hemospheres, hemulles, Figure 5) corresponding to Maloney’s domains; these data were compiled in the review [109].

From the perspective of the continuous hierarchy of biological tissues, niches should be structural and functional elements in a puzzle of larger domains. For example, His in BM have different sizes. Approximately 40% of HIs are 20–25 µm in diameter (~5–10 cells); in turn, 60% of cluster sizes are 60–80 μm on average (up to 100 cells) [56]. These dimensions may be, respectively, classified as HSPC niches or (micro)domains composed of single-cell niches [109].

Such experiments and fundamental considerations are not only of theoretical importance for the development of the niche concept at the present stage. Domain-like structures can be designed ex vivo using state-of-the-art solutions such as spheroid techniques (e.g., [159]) and various additive methods, primarily lithographic processing [160].

Regarding the need for bioengineering to mimic natural niches, there are only sporadic articles in the Scopus, PubMed, Web of Science, and Science Direct databases. For instance, Santos Rosalem et al. (2022) found only 21 research papers published between 2009 and 2020 [161,162]. Secondly, the publications share a major drawback: the authors attempt to design ‘niches’ in the broadest sense of the word, i.e., as a “dimensionless” or stochastic in its size stem cell microenvironment.

Thus, HIs and megakaryocytic niche candidates for HSPCs (Figure 5), to a certain extent also hemosheres with small sizes (~30–100 µm) [58], as well as MSC ‘osteogenic’ niches (Figure 3, Table S2), have a specific range of quite measurable parameters (size, geometry, composition) for their real reproduction in the synthetic version of the natural microenvironment.

While the morphofunctional and topographical intricacies of stem cell niches in BM and other tissues could be studied indefinitely, advancing the niche concept to quantitative and 3D bioengineering stages [13] requires a strong consensus among researchers. A clear agreement on the key challenges and well-defined milestones (Table 1) is essential to guide future progress in this field.

To gradually bridge the gap between researchers in the complex niche problematics, we developed and proposed an expert questionnaire (Table S1) covering, to some initial extent, the definition, topography, hierarchy, dimension, geometry, composition, and regulatory mechanisms of the stem cell niche. This pilot survey, being conducted under the auspices of the National Society for Regenerative Medicine in the Russian Federation, aims to establish a unified framework of niche understanding on the eve of the 50th anniversary of Schofield’s hypothesis.

## 5. Prospects for Further Research

High expectations for the determination of spatial distribution of HSC, MSC and cancer cell niches in tissues, as well as their functioning mechanisms, are laying on the latest approaches including ex vivo modeling (e.g., see [54,109,163,164,165,166], live imaging (see Section 3.4), single-cell omics (genomics, transcriptomics, proteomics, and metabolomics) techniques, and their combination. A query ‘stem cell niches and omics’ showed 72 papers in the Pubmed database since 2012, reflecting the importance of these approaches in the field of spatial molecular and cellular processes at a single-cell and multicellular levels. Single-cell RNA-sequencing, spatial transcriptomics, epigenomics, and multi-omics combined with in vivo lineage-tracing estimation within highly complex and plastic BM structure have identified multicellular diversity, growth, and regeneration of hematopoietic and non-hematopoietic cells [166,167].

Mapping the hematopoietic, stromal, and tumor cells allows designing the tissue atlases and big data-based profiles revealing the localization, fate heterogeneity, and intercellular communication of HSCs, MSCs, as well as leukemia cells in human BM [112]. There was an in situ transformation of HIM stromal elements contacted with leukemic blast cells in leukemia patients [40,112]. Certainly, the combination of live imaging, single-cell omics, and bioinformatics provides novel clinical tools to localize the pathological BM microenvironment [168,169] for improving early diagnostics, predicting and curing malignancies relapse. Age-related and pathological changes in BM niches can contribute to HSPC functional aging [40,124] or even its leukemic transformation and progression [40,170]. Therefore, anti-leukemic reprogramming or remodeling of the specific HIM [171], as well as a transplantation of young niche components [172] seems at first sight to be a fantastic clinical perspective for revitalizing aged or compromised hematopoiesis. However, HIs are real candidate niches for HSPCs (see Section 3.1) if expert consensus is reached. HIs are obviously capable of ex vivo self-assembly and subsequent transplantation; moreover, their stromal and hematopoietic elements may be precisely reprogrammed using omics technologies.

Thus, these advanced single-cell tools and techniques allow for the localization and differentiation of the micro-sites of health and pathological hematopoiesis in the HIM volume. The current evidence suggests these formations should be more accurately characterized as niche-like structures rather than definitive niches, given the persistent lack of consensus within the expert community regarding their defining characteristics. Fundamental parameters, including geometric organization, dimensional boundaries, metabolic profiles, and biomechanical properties, remain subjects of ongoing debate. Establishing expert consensus on stem cell niches represents a particularly challenging and protracted scholarly endeavor that will require coordinated, multi-stage investigation. The present review, along with the accompanying developed questionnaire (Table S1), provides a foundational framework to initiate this essential dialog. We propose that this systematic approach will enable gradual but meaningful progress toward resolving these fundamental definitional challenges that currently limit conceptual clarity in the field and restrict the clinical translation of the promising experimental results.

Importantly, a stem cell niche could be a potential therapeutic target for regenerative technologies [173]. Thus, we have recently developed a novel biopharmaceutical drug based on MSC secretome for the treatment of male infertility, building upon scientific research that elucidates the role of MSCs in the restoration of the damaged spermatogonial stem cell niche [80,173]. As another example, three-dimensional (3D) bioprinting is a promising additive (bio)technology that enables the reproducible and precise production of biological tissues and organs [174] based on living cells and natural or synthetic ECM components to mimic the hierarchical organization of multicellular populations [175]. Low-cost 3D printers allow the organization of cells and biological materials into complex scaffolds with reported resolution ~1–15 µm and average errors < 2% [176]. Current accuracy corresponds to a spatial dimension of HSC and MSC microterritories reviewed in [54,109].

Hence, niche printing may significantly improve the biomimetic properties of the scaffolds, promoting a new class of bioinspired bioengineered ECM for industrial production. Therefore, a wide-range expert consensus on the niche features can be a driver for large-scale development of a short-term strategy of 3D manufacturing for clinical applications in tissue engineering and regenerative biomedicine.

## 6. Primary Minimal Criteria for HSPC Niche

Two decades ago, the International Society for Cellular Therapy (ISCT) established some minimal criteria to define the morphofunctional properties of MSCs [177]. However, the complex properties of cells derived from the MSC pool have not yet been fully elucidated; therefore, it is probably not yet time to determine the features of their niches. Firstly, it is not always clear whether it is a niche or domain for MSCs themselves, or an MSC-derived niche for HSCs (e.g., the (peri)vascular microenvironment), or their shared specialized microspace. Secondly, even when it is obvious (i.e., chondrogenic or osteogenic niches for MSCs, Table S2), there are few data on their borders to generalize existing knowledge about the existential features of these microterritories.

At the same time, pilot experiments already need to be undertaken to discuss the quantitative parameters of HSPC niches if a common consensus is to be reached on HIs as candidates for stem and progenitor cell microterritories. It does not seem timely to summarize the properties of ‘megakaryocytic niches’ because MKs range in size (Figure 4b,d,f) from 10 µm to about 65 µm due to their significant polyploidization (up to 32N) [178,179].

Based on the above speculations, the following primary minimal quantitative and qualitative criteria for a single-cell niche candidate for HSPC can be suggested to initiate a constructive discussion in the scientific community:

- Approximately round or oval shape (Figure 4);
- Up to 5–10 mature and/or immature hematopoietic cells in close contact with stromal-derived cells [56,108];
- Niche diameter up to 100 µm, approximately [109].

## 7. Conclusions

Since R. Schofield proposed the first conception of ‘stem cell niche’, it has been considerably refined and further developed by the work of subsequent researchers. In the meantime, tissue-specific niches for multiple types of postnatal stem cells have been discovered and characterized in detail. To date, dozens of cellular and molecular components involved in stem cell regulation within the niche have been identified. However, the accumulation of this knowledge has led to a certain conceptual ambiguity and a multiplication of definitions, demanding a critical reassessment of the existing data and potentially a reformulation of the core tenets of the niche concept.

In this review, we have discussed the main recent achievements and key problems in the development of the niche concept, focusing mainly on adult mammalian HSCs and MSCs. General definitions of the stem cell niche, core niche components and regulatory mechanisms, hierarchy of potential stem and progenitor cell niche candidates, and challenges of dimension and geometry of niches were described. Some properties of erythroblastic (hematopoietic) islands as a possible niche for HSPCs, megakaryocytic niche candidate for HSPCs, and MSC niche were recognized to formulate and propose primary minimal criteria for a niche of hematopoietic stem and progenitor cells.

Finally, we have proposed three primary minimal criteria for HSPC niches (Section 6). A state-of-the-art questionnaire with six sections (Table S1) was prepared on the basis of crucial issues highlighted in the review and described briefly in Table 1. Expert surveys address the niche definition, topography, hierarchy, dimension, geometry, contribution of different niche components to regulatory function, control of cell behavior within the niche, and specific features of stem cell niches of mesenchymal origin. We plan to conduct a pilot survey using the developed questionnaire under the auspices of the National Society for Regenerative Medicine in the Russian Federation. The qualified answers, reflecting the professional positions of the experts, will be used to prepare the National Expert Consensus on the stem cell niche concept, dedicated to the 50th anniversary of R. Schofield’s hypothesis.

For this purpose, the opinions of domestic experts on each question will be collected online, and the strength of consensus on each issue will be calculated as part of the responses (agree, disagree, or abstain) of all voting experts. Further, the strength of consensus will be clarified as strong (>75%), middle (50–75%), or poor (<50%) value to validate the conclusions according to statistical analysis. Finally, the results of the National Expert Consensus will be introduced to the scientific community, and open discussion will be welcomed and developed at scientific meetings in the field.

We hope that the establishment of an up-to-date consensus is expected to further facilitate meaningful and in-depth discussions regarding the challenges and future directions of one of the most compelling issues in regenerative medicine. We anticipate that the prospective implications of our efforts will include the following key aspects:

1. Fundamental and applied scientific solutions:
  - Enhanced international research collaboration by defining clear priorities and directions;
  - Systematic development of a unified classification system for diverse structural-functional units within the native hierarchy of hematopoietic tissues, building upon previous work outlined in [101];
  - Innovative digital solutions for three-dimensional prototyping and printing of synthetic stem cell niches and other microscale tissue constructs, enabling precise bioengineering applications.
2. Educational and terminological contributions:
  - Specialized training programs to equip early-career researchers with cutting-edge knowledge in the field;
  - Development and refinement of a standardized glossary to harmonize terminology related to stem cells and their niches.

## Figures and Tables

**Table 1:** Current milestones and crucial issues of modern development of niche hypothesis for adult mammalian stem cells.

Postulates of Stem Cell Niche Hypothesis Proposed by Schofield [9,10]	Current Development of Schofield’s Hypothesis	Selective References and Sources	Crucial Issues and Relevant Questions of Niche Hypothesis Development
**General concept (definition) of the stem cell niche**			
The fundamental property of a stem cell is self-renewal, which depends on the microenvironment in which the stem cell is seen in association with other cells, determining its behavior. The cellular environment, which retains the stem cell, is a stem cell ‘niche’.	The niches are the osteal sites in the trabecular bones where CFU give rise to different lineages of hematopoiesis	[15]	There is no agreement on a common ‘niche’ definition from concept proposal to the present day.
	**Orthodox definition** A niche is a confined site (specialized microenvironment) in an organ that supports the stem cell self-renewal and maintains the HSC in a quiescent (undifferentiated) state.	[17,18,19,20,21,22]	
	**Alternative (dynamic) definition** A stem cell niche is a distinct, dynamic, hierarchical, and specialized microenvironment that provides for localization and self-renewal, regulates the balance between quiescent and proliferative states, and allows for the choice of fate and differentiation of stem cells and their progenitors.	[13,23,24,25,26,27,28]	
**Core components and regulatory mechanisms in stem cell niches**			
The microenvironment is a major component of the stem cell system. Three different supporting cell lines are required for stem cells to produce different hematopoietic lineages.	The most tangible advancement of views since Schofield’s hypothesis. Virtually all BM cells and molecules may be components of the niches for stem and progenitor cells.	Figure 1 [13,29,30,31,32,33,34,35,36]	There is no clear understanding of how a local niche is fundamentally different from the formal microenvironment of a stem cell within a specific tissue compartment [37]. This issue is particularly relevant for the stem cells of mesenchymal origin. If a cell niche is a specific anatomical microterritory, spatial restrictions should exist. Otherwise, the molecular and cellular regulatory mechanisms in the niche are not distinguished from those observed in the total HIM (Figure 2). At least, minimal quantitative and qualitative criteria are required for a stem cell niche composition, specifically the HSC, HSPC and MSC niches in adult mammals.
**Hierarchy of potential niche-candidates for stem and progenitor cells. Topographical distribution**			
Stem and progenitor cells can occupy a vacant niche in which they become a stem cell, i.e., they stop their commitment, but their capacity for self-renewal is reduced.	The classical model of hematopoiesis is hierarchical. Therefore, there can also be a niche hierarchy. According to Schofield’s hypothesis, outside the niche, the HSC begins to commit (i.e., becomes a progenitor cell); if the progenitor cell finds another niche, it can return to the ‘quiescent’ state. In this way, a hierarchy of niches emerges, as there are microterritories for both true HSCs and their progenitor cells. Indeed, many niche options have been proposed for HSCs, MSCs, and their progenies. There are numerous empty HSC niches in the BM unoccupied by transplanted HSCs. HSC niches are abundant and are not a limiting factor in vivo.	Figure 3 (Table S2) [38,39,40]	To define that stem cells have found a new niche, it is critically important to know the boundaries of niche space.
Their progeny, unless they can occupy a similar stem cell ‘niche’, are first-generation colony-forming cells that proliferate and mature to acquire a high probability of differentiation, i.e., they have an age-structure	There are young and old niches that regulate the ‘aging’ of stem cells. It is assumed that niches for hematopoietic progenitor cells exist within the HIM, in which they proliferate, differentiate, and mature. True anatomical (structural and functional) candidate niches for HSPCs are erythroblastic islands and megakaryocytes. MSCs have an ex vivo reproducible candidate niche whose space they preferentially differentiate into osteoblasts.	Table S2 [26,40,41,42,43,44,45]	To understand that the stem cell has moved out of its niche, we need to clearly define the niche borders.
The fundamental property of a stem cell is self-renewal.	In addition to the sites supporting self-renewal stem cells (quiescent niches?), active (activated) niches for hematopoietic precursor cells have been proposed, for example hematopoietic islands (HIs) (Figure 4). Multiple cellular and molecular niche signals that maintain the stem cell pool in a specific functional state have already been identified.	Figure 1 Table S2 [13,46,47]	To what extent do the intercellular interactions and cellular-molecular signals of specialized niches differ from those in the entire HIM? Are there qualitative differences in the signals or in their high concentration in a limited niche volume? To highlight, knowledge of the finite boundaries and dimensions of stem microterritories is required.
The hematopoietic niches are in close contact with the bone.	There are many variations in topographical, anatomical, and functional candidate niches for HSPCs and MSCs that are visualized in the BM.	Table S2 [48,49,50]	The location of a stem cell in a tissue site is not sufficient to define its niche, as the niche must have both anatomical and functional characteristics [26]. For example, HSPCs and MSCs are often in close contact in the BM. The question arises: whether this is an MSC-derived niche for HSPC or the MSC niche itself? This could be particularly related to the niche found by Newton in the epiphyseal growth plate [50], as the relationship between hematopoiesis, chondropoiesis, and enchondral ossification is well known [51].
**Challenges of dimension and geometry of stem and progenitor cell niches**			
Every stem cell niche is occupied. The cells may not have enough space in the niche.	It follows from Schofield’s postulate that a niche has a certain size. However, HSC territories are now considered as micro-niches, niches themselves, as well as macro-niches with a size corresponding to the entire BM. At the same time, erythroblastic islands and megakaryocytic niches for HSPCs have definitive dimensions that can be determined on cytologic and histologic preparations. Furthermore, ex vivo modeling of individual MSC niches demonstrates a preferable size range for enhancing osteogenic differentiation.	Figure 2 [40,42,44,52,53,54]	There is still no generally accepted, clear meaning of the niche boundaries (the shape and size of the stem microterritories).
Stem cells are dependent on their microenvironment.	In addition to the hypothetical niches, large multicellular associations (hematopoietic islands, domains, hematons, hemospheres, hemmules) have been experimentally defined in the BM, reflecting its structural and functional hierarchy as a hematopoietic organ.	Figure 5 [52,55,56,57,58,59]	What is the place of niches in the structural and functional organization of the BM? Are they components of larger cellular (tissue) associations, or can the entire HIM be considered a ‘niche’ with wide variations in its size?

## Data Availability

Table S1 is available on request.

## Supplementary Materials

The following supporting information can be downloaded at https://www.mdpi.com/article/10.3390/ijms26178422/s1. Table S1 and Table S2 are supplied.

## Glossary

The following definitions are used in this manuscript:

HIM	The microenvironment of hematopoietic tissues (bone marrow, spleen, and liver in rodents); its morphological components (stromal cells, ECM, blood vessels, and nerve terminals) control the hematopoietic cell fate via direct (cell–cell and cell–matrix) and indirect (soluble molecules) signals.
Niche	No common definition exists. The simplest definition is that the niche is the specific microenvironment of a stem cell. Our description of the niche is as follows: the stem cell niches are the distinct, structural-functional, energetically favorable microterritories (structural-functional regions) in a contiguous spatial space of HIM where quantitative parameters of a microenvironment promote the qualitative control of stem cell fate.
Domain	HSC regulatory volume in mouse bone marrow, with the value of ~0.1 mm^3^, consisted of ~90,000 cells (stem, progenitor, and mature hematopoietic cells + HIM components) [55,109].

## Abbreviations

The following abbreviations are used in this manuscript:

HSCs	Hematopoietic stem cells
HIM	Hemopoietic-inductive microenvironment
HER	Hemopoiesis engendered randomly
MSCs	Mesenchymal stromal/stem cells
BM	Bone marrow
HSPCs	Hematopoietic stem and progenitor cells
CFU	Colony-forming units
ECM	Extracellular matrix
LepR+	leptin receptor-expressing (cells)
SSCs	Skeletal stem cells
CAR	CXC chemokine ligand 12 (CXCL12)-abundant reticular cells
SCF	Stem cell factor
BMP	Bone morphogenetic protein
TGF	Transforming growth factor
SDF-1	Stromal cell-derived factor 1
FGF-4	Fibroblast growth factor 4
VEGF	Vascular endothelial growth factor
MMP	Metalloproteinase
KitL	Kit ligand
LGR5	Leucine-rich repeat-containing G-protein-coupled receptor 5
EGF	Epidermal growth factor
DLL4	Delta-like protein 4
FGFs	Fibroblast growth factors
EIs	Erythroblastic islands
HIs	Hematopoietic islands
MKs	Megakaryocytes
SSPCs	Skeletal stem and progenitor cells
MLM	Multiphoton laser microscopy
ISCT	International Society for Cellular Therapy
