# Supplementary material for: Stem Cell Niche Concept: Search for Current Expert Consensus
Source: Int J Mol Sci. 2025 Aug 29;26(17):8422. doi: 10.3390/ijms26178422 (PMC12429054; doi:10.3390/ijms26178422)
Supplement: Supplementary file 1 [file ijms-26-08422-s001.zip › Table S1_questionnaire_R2.pdf]

Table S1. Expert survey form to assess the status of the niche concept in the upcoming 50th anniversary of the Schofield hypothesis for postnatal mammalian cells.

No.	Discussion question/statement	Voting results			Consensus strength*	Comments
		Agree	Disagree	Abstained		
**Section A**	**General issues and niche hierarchy for stem and progenitor cells**					
A.1	The niche is a functional unit of the cellular microenvironment					
A.2	The niche is a morphofunctional unit (specialized microterritory) of the cellular microenvironment					
A.3	There is a hierarchy of stem and progenitor cells					
A.4	There is a hierarchy of the niches for stem and progenitor cells					
A.5	There is a functional hierarchy of the niches for stem and progenitor cells					
A.6	There are structural subtypes of the niches for stem and progenitor cells					
A.7	There is a hierarchy of the niches for stem and progenitor cells of various maturation and/or differentiation					
A.8	There are topographic variations in the location of stem and progenitor cell niches					
A.9	The niche is the morphofunctional unit of cell homeostasis maintenance and postnatal tissue regeneration					
**Section B**	**Issues in the topography of stem and progenitor cell niches**					
B.1	The niches in the tissues are arranged randomly					
B.2	Niches are closely related to the topography of the cell microenvironment					
B.3	The niches can change their topographic location in the tissue (niche ‘dynamism’)					
B.4	The extracellular matrix (ECM) of the					

	microenvironment is a component of the stem and progenitor cell niche					
B.5	The physical, mechanical and chemical properties of the ECM affect the niche space and functioning					
**Section C**	**Problems of dimensionality and geometry of stem and progenitor cell niches**					
C.1	There are niches for single stem cells					
C.2	There are niches for several stem and/or progenitor cells					
C.3	There are multicellular niches					
C.4	The niche has certain boundaries and size range					
C.5	The niche dimension for a single stem cell is limited to 5-10 parenchymal and stromal cells					
C.6	Niche size influences the status of stem and progenitor cells					
C.7	Niches have an ordered geometry (regular shape)					
C.8	Niches have undefined geometry (random shape)					
**Section D**	**Control of cell behavior and fate in the niche**					
D.1	The functional state of cells in the niche is regulated by a set of cues of different nature					
D.2	Stem cell control in the niche is driven predominantly by neurohumoral signals					
D.3	Stem cell control in the niche is conditioned predominantly by intercellular contacts					
D.4	Stem cell control in the niche is driven predominantly by (bio)physical and mechanical signals					
D.5	Stem cell control in the niche is driven predominantly by (bio)chemical cues and intercellular contacts					

D.6	The intensity (power) of signaling within a niche is higher than in the surrounded microenvironment					
D.7	The ability of stem cells within the niche to respond to various signals is greater than that of specialized cells					
D.8	The niche modulates systemic signals by converting them into localized stimuli					
D.9	Stem cell emigration from the niche necessarily triggers its differentiation and loss of stemness					
D.10	Cell immigration into a niche (under the control of niche signaling), including differentiated cells, can cause them to acquire stem cell properties					
D.11	Controlling cell niche behavior is a promising approach to develop new treatments for chronic diseases and injuries					
**Section E**	**Stem cell niches of mesenchymal origin**					
E.1	There are endosteal niches for hematopoietic stem cells (HSCs)					
E.2	There are (peri)vascular niches for HSCs					
E.3	Hematopoietic islands are specialized niches of HSCs and/or progenitor cells					
E.4	There are niches for mesenchymal stromal/stem cells (MSCs)					
E.5	MSC niche is perivascular					
E.6	There are other subtypes of MSC niches					
E.7	Niches can direct the MSC differentiation					
E.8	MSC plasticity is controlled predominantly genetically					
E.9	MSC plasticity is modulated predominantly by epigenetic niche signals					

Section F	Issues of compartmentalization of cellular microenvironment, tissues and organs					
F.1	There are other (except niches) cellular associations in the microenvironment space					
F.2	There is a domain organization of the tissue microenvironment based on multicellular (sub)populations					
F.3	Cell spheroids and other multicellular constructs could serve as niches for stem cells					
F.4	Embryonic stem cells able to create their own niches *ex vivo*					
F.5	There are structural-functional (anatomical) units of tissues and organs					

*The strength of consensus on each issue is determined by a vote of all experts.
