# Supplementary material for: Stem Cell Niche Concept: Search for Current Expert Consensus
Source: Int J Mol Sci. 2025 Aug 29;26(17):8422. doi: 10.3390/ijms26178422 (PMC12429054; doi:10.3390/ijms26178422)
Supplement: Supplementary file 1 [file ijms-26-08422-s001.zip › Table S2_R2.pdf]

Table S2. HSPC and MSC candidate niches in the bone marrow of adult mammalians.

Niche name	Niche-forming cells and/or functioning conditions	References
**Morphological (anatomical, structural-functional) and topographical subtypes of hematopoietic stem and progenitor cell (HSPC) niche**		
Hard or soft	Osteoblasts or endothelial cells (ECs)	[110]
Trabecular (endosteal) or (peri)vascular niches	Osteoblasts or ECs	[46,48,49,111]
Arterio-endosteal niche	A relatively hyperoxygenated site for early myelopoiesis found with the single-cell RNA sequencing	[112]
Arteriolar or sinusoidal niches	Quiescent HSCs locate closely to small arterioles near the endosteal surface.<br>Arteriolar/ sinusoidal niche maintains more quiescent HSC compared with megakaryocyte niche.	[61,113,114]
Stromal niche	Niches to support the HSPC maintaining, supporting, and regeneration in the bone marrow.	[115]
Adipocytic niche	Neighborhood for early hematopoietic stem and progenitor cell functioning detected with the single-cell RNA sequencing	[112]
Hematopoietic (erythroblastic) islands	Centrally located stromal elements (CD169+ macrophages or fibroblast-like reticular cells) surrounded by 5-30 hematopoietic cells	[42,52,56,116,117]
Megakaryocytic niche	Quiescent HSCs in direct contact with megakaryocyte which secrets TGFβ1	[44,118]
Hematopoietic stem and progenitor cell (HSPC) versus lymphoid precursor niches	Immature MSCs maintain HSPCs while more differentiated osteo-progenitors and osteoblasts support the lymphoid precursors	[113]
B cell niches versus T cell	Osx+ osteolineage cells create micro-niches for B cell development by producing IGF1 to enable pro-B to pre-B maturation.<br>Ocn+ mature osteolineage cells affect T lineage development by specifying early T competent progenitor commitment in the bone marrow through Notch signaling.	[52]
**Functional variants of HSPC niche**		
Specialized or equivalent niches	The number of niches capable of supporting HSCs is limited (specialized niche);<br>Equivalent niche support either HSCs or progenitor cells.	[119]
Quiescent or active (activated) niches	The osteoblastic niche as a "hypoxic" niche that maintains HSCs in a quiescent (slow cycling or G0) state.<br>The vascular "oxygenic" niche provides short-term stem/progenitor hematopoietic cells, which are actively proliferating, differentiating and mobilizing in blood stream.	[13,46,47]
Hypoxic BM niche	Contains many types of niche cells, including osteoblasts (endosteal niche), ECs (vascular niche), megakaryocytes, adipocytes, mesenchymal stem cells, and so on.<br>Different niche cells secrete many growth factors and	[11]

	nutrients to regulate the HSC metabolism and stemness.	
Lymphopoietic niche for T cell	There is described a pre-thymic, BM-specific role for an activation of Notch signaling pathway in early T cell development.	[120]
Inflammatory niche	Inflammatory remodeling of stromal niches to repress normal HSCs and promote human acute myeloid leukemia.	[121]
Immunological niche	Focal physiological and pathological sites where hypoxia regulates HSCs and immune homeostasis through the control of resident immune cells.	[122]
Young and aged niches	Niche aging significantly contributes to aging by affecting stem cell activity.<br>Factors produced~~s~~ in the young stem cell niche enhance progenitor cell proliferation in old mice.<br>While aging, some BM niche structures are degenerated and negatively impact HSC functionality; however, other niche cells and specific signals are preserved and essential to retaining HSC function and regenerative capacity.	[26,40,41,123,124]
**Morphological (anatomical, structural-functional) and topographical subtypes of MSC niche**		
Epiphyseal or periosteal niches	Skeletal stem and progenitor cell (SSPC) niche develops postnatally in the epiphyseal growth plate, which provides a continuous production of chondrocytes over a prolonged period.<br>There is also proposed the periosteal niche for SSPCs.	[28,50]
Microvascular niche	Self-renewing multipotent skeletal progenitors (skeletal stem cells, also known as mesenchymal stem cells) have their own microvascular niches	[125]
Perivascular niche	There is increasing evidence suggesting that MSCs may stay in the microvessel's adventitia. For all this, MSCs may serve as the progenitor cells of perivascular populations, including pericytes.	[126–128]
Vascular niche for MSCs	Arterial cells provide unique cues to regulate MSC subpopulations and maintain their heterogeneity.	[129,130]
Microenvironmental niche	Cellular structure and molecular pathways that is involved in regulation of MSC self-renewal versus differentiation, as well in retention of MSCs within the niche versus their mobilization and recruitment to sites of injury.	[131]
**Functional variants of MSC niche**		
Remodeling niche	Dynamism and flexibility of the niches reflecting bone, bone marrow stroma and hematopoiesis remodeling throughout life.	[13,125,132]
Hypoxic niche	A gradient of oxygen concentrations within multicellular associations generates hypoxic conditions that can effectively activate several signaling pathways, ultimately leading to the metabolic reconfiguration of MSCs.	[133,134]
Injury-induced stem cell niche	It regulates the proliferation and osteogenic differentiation of MSCs in conditions of heterotopic ossification. Progenitor/stem cells, vasculature, neurites, macrophages, and mast cells are closely	[135]

	associated with the proposed niche and thus are possible candidate niche supportive cells.	
Osteogenic niches for single cells	Calcium phosphate microterritories with established geometry and size preferable by human MSC-like cells to differentiate into osteoblasts.	[43,54,136]
Chondrogenic niche for skeletal stem and progenitor cells	Topographical sites in mouse epiphyseal growth plate that provide a continuous chondrocyte genesis over a prolonged period	[50]

BM – bone marrow; ECs – endothelial cells; HSCs – hematopoietic stem cells; HSPCs – hematopoietic stem and progenitor cell; MSCs – mesenchymal stromal/stem cells; IGF1 – insulin-like growth factor 1; SSPCs – skeletal stem and progenitor cells; TGFβ1 – transforming growth factor 1.
